# *KRAS* and *BRAF* genotyping of synchronous colorectal carcinomas

**DOI:** 10.3892/ol.2014.1905

**Published:** 2014-02-21

**Authors:** RICCARDO GIANNINI, CRISTIANA LUPI, FOTIOS LOUPAKIS, ADELE SERVADIO, CHIARA CREMOLINI, ELISA SENSI, MASSIMO CHIARUGI, CARLOTTA ANTONIOTTI, FULVIO BASOLO, ALFREDO FALCONE, GABRIELLA FONTANINI

**Affiliations:** 1 Department of Surgery, University of Pisa, Pisa I-56126, Italy; 2 Unit of Surgical Pathology 3, Pisa University Hospital, Pisa I-56126, Italy; 3 Unit of Medical Oncology, Pisa University Hospital, Pisa I-56126, Italy

**Keywords:** synchronous colorectal carcinomas, *KRAS*, *BRAF*

## Abstract

v-Ki-ras2 Kirsten rat sarcoma viral oncogene homolog (*KRAS*) genotyping is required prior to anti-epidermal growth factor receptor monoclonal antibody therapy administered in cases of metastatic colorectal carcinoma (CRC). Thus, *KRAS* mutation screening is required for patient management. The present study reported the experience of *KRAS*/v-raf murine sarcoma viral oncogene homolog B1 (*BRAF*) mutational screening on synchronous CRC pairs from 26 patients, which were defined as index lesions (ILs) and concurrent lesions (CLs) on the basis of tumor grade and dimension and their respective lymph node and distant metastases. Overall, *KRAS* mutations were present in 38.4% of patients, whereas *BRAF* mutations were present at a frequency of 11.5%. The genotyping of paired synchronous carcinomas indicated that 11 patients (42.3%) exhibited discordant *KRAS* mutational statuses in terms of the presence of a mutation in only one lesion of the pair or of two different mutations harbored by each lesion. *BRAF* mutations were present in the synchronous tumors of two cases, whereas in two other cases, only the IL or CL harbored mutant *BRAF.* Overall, the mutational statuses of distant and lymph node metastases confirm the genetic heterogeneity of synchronous primary tumors. These results highlighted the fact that adequate sampling and comprehensive testing, when feasible, is likely to optimize the decision-making process for treatment approaches, even in the relatively rare event of multiple synchronous lesions.

## Introduction

Two monoclonal antibodies, cetuximab and panitumumab, which target the epidermal growth factor receptor (EGFR), were approved in Europe and the United States for the treatment of metastatic colorectal carcinoma (CRC) in 2004 and 2007, respectively. Subsequently, somatic gain-of-function v-Ki-ras2 Kirsten rat sarcoma viral oncogene homolog (*KRAS*) [and v-raf murine sarcoma viral oncogene homolog B1 (*BRAF*)] mutations have been identified as reliable and strong negative predictors for the response to anti-EGFR treatment in CRC (1–6). These specifically include point mutations located in codons 12 and 13, which represent ~98% of all *KRAS* mutations in CRC. Based on these observations, the European Medicines Agency has curtailed the application of palliative cetuximab and panitumumab therapies for the treatment of CRC depending on the *KRAS* wild-type status of the tumor tissue, regardless of whether the antibodies are applied in combination with conventional chemotherapy or as single agents. BRAF, which is downstream from KRAS in the mitogen-activated protein kinase pathway, is subject to activating mutations that facilitate the development of resistance to EGFR-targeted monoclonal antibody treatments (7,8).

Several assays for *KRAS* and *BRAF* mutations have been developed that involve DNA extraction from a single tumor tissue block, followed by a mutation-specific, polymerase chain reaction (PCR)-based assay or the sequencing of the relevant codons. The selection of the tissue blocks for analysis is of clinical relevance for the following reasons: i) The tissue sampled for genotyping may contain a small population of malignant cells and a large population of stromal and inflammatory cells; ii) the potential genetic heterogeneity of the tumor tissues in terms of *KRAS* and/or *BRAF* mutational status; and iii) the genetic heterogeneity of multiple types of colorectal cancer may result from one or both of the above-mentioned circumstances or from an independent tumor origin. Thus, investigations that assess the genomic alterations and degrees of similarity or differences in multiple types of synchronous cancer may provide insight into more opportune treatments in the presence of multiple neoplasms. (9–14)

Multiple primary carcinomas often occur in the large intestine, and the time to malignant transformation is variable. Synchronous carcinomas (SC) are defined as two or more primary carcinomas that coexist at the time of diagnosis or that are diagnosed within the same six-month period. SCs exhibit an incidence of 3–5% and a poorer prognosis, with a significantly increased risk of distant metastases compared with solitary CRCs. Although, the prognostic significance of cancer synchronicity remains unclear (15–18).

From a molecular standpoint, the theoretical basis of ‘Colorectal Adenoma and Cancer Divergence’ was previously proposed by Tsao *et al* in 1999 (19). Recently, Balschun *et al* analyzed the genotypes of the *KRAS*, neuroblastoma RAS viral (v-ras) oncogene homolog, phosphatidylinositol-4,5-bisphosphate 3-kinase, catalytic subunit α (*PIK3CA) exon 20* and *BRAF* genes in synchronous and metachronous primary CRCs and reported a certain grade of heterogeneity in synchronous CRC (SCRC) patients (65%). The study further discussed the diagnostic and therapeutic implications of these observations, recommending ‘that therapy should be tailored by the genotype of the lesion to be treated’ (20).

The present study investigated the frequency and distribution of *KRAS* and *BRAF* mutations in primary tumors and the lymph node and distant metastases of 26 patients with SCRCs using highly sensitive and specific methods, both in terms of sample handling and mutational analysis.

## Materials and methods

### Patients and samples

In total, 26 patients with SCRC were selected from a consecutive series of 500 cases derived from the patient files of Pisa University Hospital (Pisa, Italy) between January, 2006 and March, 2010. The study was approved by the Institutional Review Board of the University of Pisa, (Pisa, Italy). All patients provided their informed consent.

All the cases were retrospectively reviewed by two pathologists and the tumor stages were determined according to the TNM classification (TNM Classification of Malignant Tumors, International Union Against Cancer, 7th edition) (21).

All the selected patients presented with two adenocarcinomas that were grossly, unequivocally separated by normal colorectal mucosa at the initial diagnosis of the CRC. The average distance between the two tumors was 25.11±2.2 cm (minimum, 1 cm; maximum, 64 cm). Among the carcinomas, the index lesions (ILs) were defined as the tumors that were the most pathologically advanced or the largest, whereas the other lesions were designated as the concurrent lesions (CLs) (9,10). Clinicopathological characteristics of the analyzed SCs are summarized in Table I.

Lymph node metastases were reported in 18 patients, of which eight also presented with distant metastases. One patient presented only with distant metastases. In the case of multiple lymph node and/or distant metastases, each reported and/or available metastatic node or distant metastasis was analyzed.

### Microdissection and DNA extraction

A total of 77 formalin-fixed, paraffin-embedded (FFPE) tissues were collected and serially cut into four 10-μm thick sections. The last section was stained with hematoxylin-eosin and the area of each tumor was delineated by a pathologist. The following samples were analyzed in further detail: 26 ILs; 26 CLs; 17 lymph node metastases; and 8 distant metastases.

Next, the tumor cells were manually microdissected from three unstained sections, transferred into 180 μl ATL buffer and digested with 20 μl proteinase K overnight at 56°C. DNA isolation was performed according to the FFPE tissue instructions recommended by the QIAamp DNA Mini kit (Qiagen, Valencia, CA, USA).

The nucleic acids were eluted in 40 μl AE buffer and the DNA content was measured using a NanoDrop-1000 spectrophotometer (Thermo Fisher Scientific, Waltham, MA, USA).

### Detection of KRAS and BRAF mutations by pyrosequencing

A total of 5 μl genomic DNA (20 ng/μl) was amplified using anti-EGFR Moab response^®^ (*KRAS* and *BRAF* status) conformité Européene-in vitro diagnostic (CE-IVD)-marked kits (Diatech Pharmacogenetics Srl, Jesi, Italy) on a Rotor-Gene^TM^ 6000 (Corbett Research, Sydney, Australia), according to the manufacturer’s instructions. The resulting PCR product was immobilized onto magnetic streptavidin-coated beads (Diatech Pharmacogenetics Srl) via biotin/streptavidin interaction. The bead/DNA complexes were then washed and added to 1.65 pmol pyrosequencing primer, which was included in the same kit. The primed, single-stranded DNA templates were transferred to a microtiter plate-based PSQ HS 96A Pyrosequencer (Biotage AB, Uppsala, Sweden) in which the real-time sequencing of the sequence surrounding codons 12 and 13 of *KRAS* and codon 600 of *BRAF* was performed using PyroMark Gold Q96 reagents (Qiagen) on a PyroMark^TM^ Q96 ID instrument (Biotage AB). The results were analyzed using PyroMark Q24 1.0.9 software.

## Results

### KRAS and BRAF genotyping

Overall, *KRAS* mutations were detected in 14 of the 26 SC patients (53.8%), whereas *BRAF* mutations were found in only four patients (15.4%).

### KRAS/BRAF mutational status of the ILs

A total of 10 *KRAS* mutations were detected among the 26 ILs, with a mutation rate of 38.5%. In particular, 8 of the 10 mutations were in *KRAS* codon 12, whereas two were in codon 13. In total, three ILs (11.5%) harbored *BRAF* mutations (Table II).

### KRAS/BRAF mutational status of the CLs

*KRAS* mutations were detected in 10 (38.5%) of the CLs; seven were located in *KRAS* codon 12 and three were in codon 13. In total, three CLs (11.5%) harbored *BRAF* mutations (Table II).

### KRAS/BRAF mutational status of the lymph node and distant metastases

A total of six *KRAS* mutations were found and analyzed in the lymph node, with four in the distant metastases. By contrast, three *BRAF* mutations were found in the lymph node, with one in the distant metastases (Table II).

### Distribution of KRAS/BRAF mutations among the ILs and CLs. KRAS mutational status

Among the 26 SCs tested, a total of 15 cases (57.7%) exhibited the same genotype (genetically homogeneous), whereas, 11 (42.3%) exhibited a different genotype (genetically heterogeneous). In total, 12 of the 15 homogeneous cases harbored wild-type *KRAS* and two cases exhibited the same mutation in each tumor. Among the *KRAS* heterogeneous cases, eight exhibited a discordant *KRAS* mutational status between the two lesions, and three harbored a different mutation (Table II).

### BRAF mutational status

In total, 24 cases were genetically homogeneous, whereas two (7.7%) were heterogeneous. Among the homogeneous pairs, two harbored the V600E mutation in the two lesions and 22 harbored wild-type *BRAF* (Table II).

### Distribution of KRAS/BRAF mutations between the ILs, CLs and distant and lymph node metastases

Genotype concordance between the ILs and relative distant metastases was found in all the analyzed cases, whereas in five patients, the CLs exhibited a different *KRAS* mutational status compared with that of the distant metastases (Table II). A single patient with a mutant *BRAF*-harboring distant metastasis harbored the same mutation as in the IL, but not the CL (Table II).

The lymph node metastasis *KRAS* genotype corresponded with that of the IL in 12 out of 14 patients. In one patient, two lymph node metastases were found that harbored two different *KRAS* mutations, which corresponded with that of the IL and CL, respectively. In one patient, lymph node metastasis was found harboring the same *KRAS* mutation as that in the CL (Table II).

## Discussion

*KRAS* genotyping is required prior to anti-EGFR monoclonal antibody therapy that is administered in cases of metastatic CRC. Thus, *KRAS* mutation screening is required for patient management (1–6). Screening of the *BRAF* mutational status is not yet a prerequisite for the administration of anti-EGFR treatments, but may become required by clinicians, given its strong prognostic significance (7,22).

The current study reported the experience of *KRAS*/*BRAF* mutational screening performed on 26 FFPE SC pairs and their respective lymph node and distant metastases.

*KRAS* mutations were present in 38.5% of the ILs and CLs, whereas BRAF mutations were present at a frequency of 11.5% in the ILs and CLs.

The *KRAS* and *BRAF* mutational rates for the ILs and CLs are in line with those previously reported by Balschun *et al* in synchronous and metasynchronous CRCs (20). In the present series, the genotyping of paired SC tumors indicated that 11 patients (42.3%) exhibited discordant *KRAS* mutational statuses in terms of the presence of a mutation in only one lesion of the pair or of two different mutations harbored by each lesion. By contrast, 15 patients exhibited the same *KRAS* genotype; three patients harbored the same *KRAS* mutation as in the IL and CL, whereas 12 harbored wild-type *KRAS. BRAF* mutations were present in the ILs and CLs of two cases, whereas in two other cases, only the IL or CL harbored mutant *BRAF*.

Intratumorally, *KRAS* and *BRAF* mutations are mutually exclusive. No single tumor was identified that harbored *KRAS* and *BRAF* mutations. Notably, *KRAS* and *BRAF* mutations were not mutually exclusive when considering SC pairs. One patient was identified with a mutant KRAS-harboring IL with a mutant BRAF-harboring CL and an additional patient exhibited the inverse distribution of these mutations. Furthermore, the *KRAS* and *BRAF* genotype was not found to correlate with increased T-status or grade of the carcinomas.

In the current series, distant metastatic lesions were reported in nine patients, of which tissue samples for genotyping were available from eight. Lymph node metastases were reported in 18 patients, of which tissue samples were available from 14 (Table II). Overall, the mutational statuses of distant and lymph node metastases confirm the genetic heterogeneity of synchronous primary tumors.

In particular, the present study found that the genotype of the distant metastases is always the same as that of the IL, and in three cases, the same as that of the CL. Among the five heterogeneous cases, three harbored different mutations in the IL and metastasis compared with the CL. Notably, in two patients, the distant metastases and IL were wild-type, whereas the CL harbored mutant *KRAS*.

Consequently, the genetic analysis of metastatic SCs may fail in predictive value due to the heterogeneous *KRAS* mutational status of the IL and CL. Taken together, the results of present study confirmed the genetic heterogeneity of *KRAS* and *BRAF* that was previously reported by Balschun *et al.* This previous study suggested that an examination of an arbitrarily selected archival tumor sample may carry the risk of a non-representative genotype and thus, of inadequate treatment (20). These observations partially contradict the observations by Baldus *et al* (23) and Voutsina *et al* (24). In particular, Baldus *et al* studied *KRAS, BRAF* and *PIK3CA* distribution patterns in primary tumors and corresponding metastases and suggested that from a diagnostic perspective, genotyping ‘should preferentially be done on samples of primary tumors or distant metastases, whereas lymph node metastases seem to be a less appropriate tool’ (23). In studying *KRAS*, *PIK3CA* and *BRAF* mutations together with MET and PTEN expression in colorectal primary tumors and corresponding metastases, Voutsina *et al* more recently concluded that metastatic lesions are the most appropriate tissues to analyze in order to determine the appropriate targeted therapies in metastatic CRC. This is despite discordance between the primary CRC tumors and associated metastases in terms of the biomarkers examined, with the exception of *BRAF* mutations (24).

From a practical perspective, these results highlight the requirement for a tight cooperation between oncologists and pathologists when assessing *KRAS* and *BRAF* mutational status for clinical purposes. Adequate sampling and comprehensive testing, when feasible, is likely to optimize the decision-making process for treatment approaches, even in the relatively rare event of multiple synchronous lesions.

## Figures and Tables

**Figure 1:**
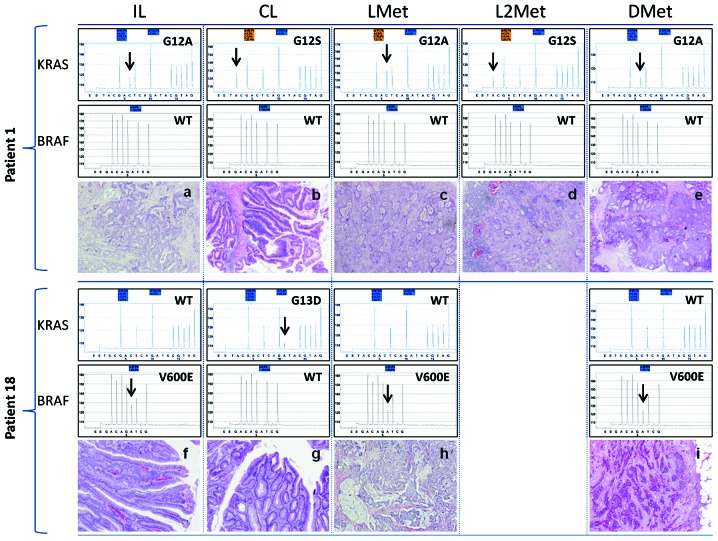
Schematic representation of hematoxylin and eosin-stained sections and relative pyrograms of two patients. The upper lane presents the KRAS analysis, the middle lane presents the BRAF analysis and the lower lane presents the histology in patients 1 and 18 (magnification, ×20). (a) Patient 1 index lesion: moderately differentiated adenocarcinoma. (b) Patient 1 concurrent lesion: differentiated adenocarcinoma. (c and d) Patient 1 regional lymphnode: metastases of adenocarcinoma. (e) Patient 1 omental lesion: metastases of adenocarcinoma. (f) Patient 18 index lesion: moderately differentiated adenocarcinoma. (g) Patient 18 concurrent lesion: moderately differentiated adenocarcinoma. (h) Patient 18 regional lymphnode: metastasis of adenocarcinoma. (i) Patient 18 omental lesion: metastasis of adenocarcinomas. KRAS: G12A, pyrogram trace showing a G to C mutation in position 2 of codon 12; G12S, pyrogram trace showing a G to A mutation in position 1 of codon 12; G13D, pyrogram trace showing G to A mutation in position 2 of codon 13. BRAF: V600E, pyrogram trace showing a T to A mutation in position 2 of codon 600. Arrows identify the sequence alterations. WT pyrogram trace revelas a normal genotype. Light blue and orange areas indicate the variable position. KRAS, v Ki ras2 Kirsten rat sarcoma viral oncogene homolog; BRAF, v raf murine sarcoma viral oncogene homolog B1; IL, index lesion; CL, concurrent lesion; LMet, lymph node metastases; DMet, distant metastases; WT, wild type.

**Table I:** Clinicopathological features of the SCRC pairs.

Variables	IL	CL
Lesions, n	26	26
Average size, cm (SEM)	4.91 (±1)	3 (±1)
Grade, n		
Moderate	18	20
Poor	8	6
% of mucinous component, n		
0	15	16
<50	7	8
≥50	4	2
Wall penetration, n		
pT2	5	20
pT3	19	6
pT4b	2	0

SCRC, synchronous colorectal carcinoma; IL, index lesion; CL, concurrent lesion.

**Table II:** *KRAS* and *BRAF* genotype (n=26).

	*KRAS*					*BRAF*				
		
Patient	IL	CL	LMet	L2Met	DMet	IL	CL	LMet	L2Met	DMet
1	G12A	G12S	G12A	G12S	G12A	WT	WT	WT	WT	WT
2	G12C	G12C	NP		G12C	WT	WT	NP		WT
3	G12C	WT	G12C	WT		WT	WT	WT	WT	
5	G12D	G12D	NP			WT	WT	NP		
4	G12D	WT	NP		G12D	WT	V600E	NP		WT
6	G12S	WT	WT			WT	WT	WT		
7	G12V	G12D				WT	WT			
8	G12V	G13D	G12V		G12V	WT	WT	WT		WT
9	G13D	G13D	G13D			WT	WT	WT		
10	G13D	WT	G13D			WT	WT	WT		
11	WT	G12A	WT			WT	WT	WT		
12	WT	G12D				WT	WT			
13	WT	G12D	WT		WT	WT	WT	WT		WT
18	WT	G13D	WT		WT	V600E	WT	V600E		V600E
19	WT	WT			WT	WT	WT			WT
20	WT	WT				WT	WT			
14	WT	WT				WT	WT			
15	WT	WT				WT	WT			
16	WT	WT				WT	WT			
17	WT	WT	WT		WT	WT	WT	WT		WT
21	WT	WT	WT			WT	WT	WT		
22	WT	WT	WT			WT	WT	WT		
23	WT	WT	NP		NP	WT	WT	NP		NP
24	WT	WT	WT			V600E	V600E	V600E		
25	WT	WT	WT	WT		V600E	V600E	V600E	WT	
26	WT	WT				WT	WT			

*KRAS*, v-Ki-ras2 Kirsten rat sarcoma viral oncogene homolog; *BRAF*, v-raf murine sarcoma viral oncogene homolog B1; IL, index lesion; CL, concurrent lesion; LMet, lymph node metastases; DMet, distant metastases; WT, wild-type; NP, not performed.
